# Nonlinear Effects of Economic Policy Uncertainty Shocks on Carbon Emissions in China: Evidence from Province-Level Data

**DOI:** 10.3390/ijerph192316293

**Published:** 2022-12-05

**Authors:** Chao Wu, Ziyu Liu, Jinquan Liu, Mingze Du

**Affiliations:** 1 School of Economics and Statistics, Guangzhou University, Guangzhou 510006, China; 2 School of Mathematics and Statistics, Liaoning University, Shenyang 110036, China

**Keywords:** EPU, carbon emissions, nonlinear effects, PSTR model

## Abstract

Based on cross-sectional data from 30 Chinese provinces from 2004 to 2017, this paper systematically examines the nonlinear effects of economic policy uncertainty (EPU) on carbon emissions and its causes using the PSTR model. It is found that the impact of EPU on carbon emissions at the provincial level in China has significant nonlinear characteristics and shows a positive and then negative pattern as the level of EPU increases. Furthermore, increased levels of EPU also cause a nonlinear migration of the effects of provincial economic and financial development, industrial structure, government spending, and environmental regulation on carbon emissions, illustrating a large amount of heterogeneity among Chinese provinces. Specifically, provinces with higher levels of economic and financial development experience a greater positive carbon emission effect from EPU, whereas provinces with lower levels of such development experience a greater negative carbon emission effect. In contrast, in provinces with irrational industrial structures, lower fiscal expenditures, and weaker environmental controls, the nonlinear carbon emission consequences of EPU are greater. Therefore, local governments should prudently adjust economic policies, improve and perfect the market information disclosure system, and afford full play to regional comparative advantages to help achieve the “double carbon goal”.

## 1. Introduction

The deterioration of global environmental quality and extreme natural disasters have sounded the alarm for humans to protect the environment, and the development of a low-carbon economy with “low pollution, low energy consumption, and low emissions” has become a goal for countries around the world. At the United Nations General Assembly’s 75th General Debate in 2020, China proposed increasing its national contribution, implementing more aggressive policies and measures, and aiming for peak carbon emissions by 2030 and carbon neutrality by 2060. The proposed carbon peak and carbon neutral targets, on the one hand, respond to the global trend of sustainable economic development [1,2], fully demonstrate China’s role as a great power, and also prompt a renewed energy revolution and paradigm change and further economic development in China’s “countdown”; on the other hand, these targets also guide China’s response to climate change and green low-carbon development, and set specific emission reduction targets for governments and the public at all levels.

China’s rapid economic growth relies on huge energy consumption, which makes China one of the world’s largest carbon emitters and presents a serious threat to its sustainable economic development. From carbon peaking to carbon neutrality, China will take 30 years to complete the path that Western countries set off on 60 years ago. At present, the domestic and international political and economic environment has undergone obvious changes, a prominent manifestation of which is the frequent occurrence of uncertain events. Therefore, under the carbon neutrality target, China also faces the pressure of many crises, such as trade friction, the COVID-19 pandemic, and geopolitical risks, bringing many challenges and uncertainties to China’s macroeconomic policy practices [3,4,5], which adversely affect national economic development [6] and in turn the development of and policy planning for the energy, manufacturing, and transportation sectors. Altogether, these pressures are detrimental to the achievement of China’s carbon neutrality target. As economic and ecological systems become increasingly interconnected, it is of great practical and economic importance to analyze the impact of EPU on China’s carbon emissions to promote the achievement of China’s “double carbon goal” and contribute to sustainable economic development.

This study aims to investigate the effect of EPU on carbon emissions in China and its mechanism of action, with core work and marginal contributions in two main areas. First, this paper focuses on the effect of EPU shocks on carbon emissions as explored by relevant studies around the world. While clarifying the internal logic of EPU shocks on carbon emissions, it integrates the divergent findings of previous studies and the structural differences of China’s regional economy and empirically investigates the effect of EPU shocks on carbon emissions in China from a nonlinear perspective based on the panel smooth transition regression (PSTR) model, which is a useful supplement to the past studies. Second, to build an intuitive understanding of the reasons and mechanisms behind the effects of EPU shocks on carbon emissions in China, this paper further simulates and analyzes the nonlinear carbon emission effects of EPU under different economic or financial conditions in Chinese regions. The rest of this paper is structured as follows: Section 2 reviews the relevant literature to identify the current state of research as well as recent breakthroughs; Section 3 constructs the empirical model, followed by a description of the data used in the study and the results of the parameter estimation; Section 4 presents the study’s findings; Section 5 explains and discusses the findings; and Section 6 concludes the paper.

## 2. Literature Review

Focusing on the core research question of this paper, we find that there are two mechanisms behind the impact of EPU on carbon emissions: first, EPU affects the rate of economic development and changes the intensity of energy consumption, which in turn affects carbon emissions; second, EPU directs governmental attention to the implementation of environmental protection policies, leading to changes in the intensity of environmental regulation and supervision, which ultimately affects carbon emissions.

Specifically, regarding the first mechanism of EPU shocks’ effects on carbon emissions in terms of the economic effects, Baker et al. [7] found that output, investment, and employment all decrease when EPU rises in the US. Huang and Luk [4] employed a structural vector autoregressive (SVAR) model to systematically examine China’s macroeconomic response to EPU shocks, concluding that rising EPU dampens real economic activities such as output and employment, which is consistent with findings in other economies. The environmental Kuznets curve (EKC) is a common tool for the study of economic growth-related carbon emission effects [8,9]. Scholars from different nations have empirically demonstrated that economic growth is a significant factor affecting carbon emissions using the EKC model [10,11]. As demonstrated in previous research, economic effects are indeed an important channel through which EPU shocks affect carbon emissions.

The second mechanism of EPU shocks’ effect on carbon emissions suggests that the intensity of environmental regulation may be relaxed when EPU rises and governments focus more on stabilizing economic development and overcoming the adverse effects of economic fluctuations [12,13]. However, lower regulating levels can lead to a tendency for companies to pay environmental fines and reduce investment in environmental R&D because of insufficient penalties. Instead, when uncertainty is low, the government generally increases the production costs of high-polluting enterprises through taxation and other means and promotes the green transformation of enterprises by providing financial support, at which point high-emission, high-polluting, and inefficient enterprises or industries generally decline [14]. Clearly, the severity of environmental regulation and oversight may shift as a result of EPU shocks, which could ultimately impact carbon emissions.

Further, most previous frontier studies that empirically investigated the carbon emission effects of EPU from different perspectives assumed a linear effect between EPU and carbon emissions. For instance, Jiang et al. [15] contended that increasing EPU considerably increases the carbon emissions of the United States through both direct and indirect effects on economic demand and policy moderation. Similarly, Wang et al. [16] found that US carbon emissions show a positive relationship with EPU. Based on cross-country data, Wang et al. [17] discovered that EPU increases carbon emissions, whereas economic development, globalization, and international trade significantly moderate the carbon emission effects of EPU. By contrast, there are results in the existing literature that contradict the above studies. For example, Chen et al. [18] conducted an empirical study using a panel model with data from a sample of 15 countries and discovered that EPU has a negative carbon emission effect, which is greater in emerging market countries than in developed countries. Similarly, Liu and Zhang [19] found that EPU in China has a negative impact on carbon emissions, but this relationship was not observed to be significant in the central or western regions.

Meanwhile, some studies shifted the relationship between EPU and carbon emissions from a linear to an asymmetric setting. For example, the EKC model framework was used by Odugbesan and Aghazadeh [20] to examine the impact of EPU on carbon emissions in Japan. They found that there is a long-term cointegration relationship between EPU and carbon emissions and that EPU significantly contributes to the rise in carbon emissions. Anser et al. [21] and Syed and Bouri [22] empirically identified the asymmetric carbon emission effects of EPU based on an autoregressive distributed lag (ARDL) model analytical framework, and found that in the short run, EPU exacerbates carbon emissions, suggesting that high EPU leads to environmental degradation in the short run; conversely, in the long run, EPU reduces carbon emissions, which implies that high EPU improves environmental quality over time.

There are significant structural differences between regional economies in China, such as the policy intensity and economic and financial development conditions of each region [23,24], and these differences may affect the magnitude of the response of real economic activity to EPU shocks, which in turn affects carbon emissions via economic or policy effects; these factors are also directly related to the factors that affect the magnitude of the response of actual economic activity to uncertainty shocks, as highlighted by theoretical studies. Specifically, in terms of economic structure, regions with higher concentrations of the financial and real estate industries are likely to be impacted if uncertainty shocks have a significant influence on financial markets. Manufacturing is likely to be sensitive to changes in short-term interest rates, as Dai and Lin [25] pointed out, and there is significant asymmetry and heterogeneity. If uncertainty shocks influence interest rates, their combined effect might be greater in areas with a more active manufacturing sector.

In terms of the condition of financial development, uncertainty affects the economy primarily through its impact on financial markets [26,27]; in this view, if financial market conditions are poor and financial frictions are prevalent, uncertainty shocks affect the real economy through their impact on the external financing premium, implying that their magnitude may be closely related to the intensity of financial frictions. In addition, differences in fiscal capacity across Chinese regions may lead to different responses to uncertainty shocks, implying that the degree of impact of uncertainty shocks may be influenced by the degree of support available to economic agents when their income or employment prospects decline. Furthermore, Carrière-Swallow and Céspedes [28] pointed out that local fiscal policies may also affect the amplification and transmission effects of financial frictions on uncertainty shocks across regions. Tao et al. [29] argue that financial development can directly influence carbon emissions.

Based on the abovementioned studies, it is clear that there is obvious divergence in past studies on the carbon emission effects of EPU, possibly resulting in very different research conclusions. This may be because the mechanism of EPU’s effects on carbon emissions is highly complex, and different factors, such as economic structure and economic and financial development status, may lead to significant changes in the mechanism of action of EPU on carbon emissions; thus, EPU shocks may have state-dependent nonlinear effects on carbon emissions. Therefore, previous studies based on linear VAR models or standard panel data models ignore the state-dependent effects of EPU, with the standard panel data models in particular mostly assuming cross-sectional homogeneity of slope parameters, and although heterogeneity can be controlled by adding additional regression variables, it is still difficult to explicitly characterize the cross-sectional dependence of the identification results. Furthermore, the majority of research to date has examined the national-level effects of EPU on carbon emissions without systematically examining the impact mechanisms, particularly the transmission mechanisms between shocks within a nation. It is obviously challenging to utilize the findings from research at the national level to inform the future development requirements of specific regions due to the existence of regional disparities among countries.

Thus, this paper selects the PSTR model proposed by Gonzalez et al. [30] to systematically examine the nonlinear and heterogeneous effects of provincial EPU shocks on carbon emissions in China. The PSTR model can be viewed as an improvement over the PTR model [31], with an unsmoothed variance of regression coefficients across different regimes, as well as an extension of the STAR model [32] from time series to panel data. Hence, the PSTR model has a clear advantage in examining the mechanism of nonlinear effects of provincial EPU shocks on carbon emissions in China because it can convincingly depict the nonlinear correlations among variables on the one hand and effectively capture the heterogeneous characteristics of panel data by smoothing the transformation of threshold variables across different regimes on the other. In addition, because of the structural differences in China’s regional economies, this paper includes indicators of economic development level, industrial structure, financial development status, local fiscal scale, and environmental regulation in the explanatory variables to account for economic, financial, and policy factors and enhance the precision of model identification. In summary, as a useful supplement to past studies, this paper examines the mechanism of the nonlinear effects of EPU shocks on carbon emissions using the PSTR model based on provincial panel data in China to provide useful empirical evidence and policy insights, aiming to accelerate China’s achievement of the “double carbon goal”.

## 3. Methods, Data, and Parameter Estimation

### 3.1. PSTR Model

The PSTR model proposed by González et al. [30] relaxes the assumption of linear relationships between variables, and the model not only better captures the cross-sectional heterogeneity of the panel data, but also allows for continuous and smooth nonlinear shifts in the model parameters with changes in the transformed variables, thus more closely matching economic reality. The specific expression of the PSTR model is:

(1) $$y_{it}=\alpha_{i}+\beta_{0}^{’}x_{it}+\beta_{1}^{’}x_{it}\Gamma \left(q_{it};\gamma ,c\right)+\varepsilon_{it}$$

where $y_{it}$ is the explained variable; $x_{it}$ is the set of vectors containing *k* explanatory variables; and in i=1,…,N and t=1,…,T, *N* and *T* denote the number of cross-sections and the length of time of the panel data, respectively. $\varepsilon_{it}$ is the stochastic perturbation term of the model. $\alpha_{i}$ denotes panel fixed effects, $\beta_{0}^{’}$ denotes the coefficients to be estimated for the explanatory variables in the model, and $\beta_{1}^{’}$ denotes the coefficients to be estimated for the transformation function in the model. $\Gamma \left(\cdot \right)$ is a logistic-type transformation function that is continuously bounded on the transformation variable, and its specific form can be expressed as:

(2) $$\Gamma (q_{it};\gamma ,c)={\left\{1+\exp[-\gamma \prod_{n=1}^{m}\left(q_{it}-c_{n}\right)]\right\}}^{-1}$$

where γ is the smoothing parameter of the conversion function, which determines the speed of the regime conversion or the smoothness of the adjustment; *c* is the position parameter, also known as the threshold level, which determines the location of the regime conversion; *m* reflects the number of position parameters. Having two position parameters, indicated as m=2, is generally more common than having one, indicated as m=1. Combining Equations (1) and (2), it can be seen that the PSTR model degenerates to a two-regime PTR model when m=1 and γ→∞; when m=2 and γ→∞, the PSTR model is transformed into a three-regime PTR model. If $q_{it}=c$ or γ→0, the PSTR model degenerates to a linear fixed-effects model. It can be seen that the PSTR model has a more flexible functional form compared with the PTR model; compared with the traditional fixed-effects or random-effects models, the PSTR model can effectively capture the nonlinear and heterogeneous features in the data.

Before constructing the PSTR model, it is first necessary to test whether there is a nonlinear effect in the model. If it exists, it is a PSTR model; otherwise, it is a linear fixed-effects model, and it is not necessary to build the PSTR model. Thus, the nonlinearity test is an important basis for model selection in this paper. Specifically, the null hypothesis is set as γ=0 or $\beta_{1}^{’}=0$, and the *LM*, *LMF*, and *LRT* statistics are constructed. If the null hypothesis is rejected, the model is characterized by nonlinearity and a PSTR model can be built for estimation. To effectively solve the parameter identification problem, the first-order Taylor expansion of $\Gamma (q_{it};\gamma ,c)$ at γ=0 is required in the specific test process to obtain the following auxiliary regression equation.

(3) $$y_{it}=\alpha_{i}+\beta_{0}^{’*}x_{it}+\beta_{1}^{’*}x_{it}q_{it}+\cdots +\beta_{m}^{’*}x_{it}q_{it}{}^{m}+\varepsilon_{it}$$

Obviously, the test of the null hypothesis is converted into a test of the hypothesis $\beta_{1}^{’*}=\cdots =\beta_{m}^{’*}=0$. Under this hypothesis, c and d are the residual sums of squares of the linear fixed-effects model and the two-area PSTR model, respectively. Each of the above three statistics can then be expressed as:

(4) $$LM=TN\frac{SSR_{0}-SSR_{1}}{SSR_{0}}$$

(5) $$LMF=\frac{\left(SSR_{0}-SSR_{1}\right)}{mk}/\frac{SSR_{0}}{\left(TN-N-m\left(k+1\right)\right)}$$

(6) $$LRT=-2\left[\log\left(SSR_{0}\right)-\log\left(SSR_{1}\right)\right]$$

where *T* is the time length of the panel data, *N* is the number of individuals in the cross-section, and *k* is the number of explanatory variables.

### 3.2. Model Setting

In this paper, provincial carbon emissions (CO_2_) in China are selected as the explained variable, and the core explanatory variable is provincial EPU; based on the nonlinear and heterogeneous mechanism of *EPU* shocks’ effect on carbon emissions summarized above, this paper integrates provincial economic, financial, and policy information, and selects the provincial level of economic development (RGDP), financial development condition (*FDC*), industrial structure (*IST*), fiscal expenditure (*FEX*), and environmental regulation (*ERE*) indicators as control variables. Referring to the setting of González et al. [30], the form of the PSTR model used to explore the mechanism of the impact of provincial EPU on nonlinear shocks to carbon emissions in China is set as follows:

(7) $$\begin{array}{l} CO_{2it}=\alpha_{i}+\beta_{1}EPU_{it}+\beta_{2}RGDP_{it}+\beta_{3}FDC_{it}+\beta_{4}IST_{4it}+\beta_{5}FEX_{it}+\beta_{6}ERE_{6it}\\ +(\beta_{1}^{’}EPU_{it}+\beta_{2}^{’}RGDP_{it}+\beta_{3}^{’}FDC_{it}+\beta_{4}^{’}IST_{4it}+\beta_{5}^{’}FEX_{it})\Gamma (EPU_{it};\gamma ,{\bar{EPU}}_{n})+\varepsilon_{it} \end{array}$$

(8) $$\Gamma (EPU_{it};\gamma ,{\bar{EPU}}_{n})={\left\{1+\exp[-\gamma \prod_{n=1}^{m}\left(EPU_{it}-{\bar{EPU}}_{n}\right)]\right\}}^{-1}$$

Under the above model setting, the influence coefficient $\delta_{it}$ of EPU on carbon emission can be expressed as:

(9) $$\begin{array}{l} \delta_{it}=\frac{\partial CO_{2it}}{\partial EPU_{it}}=\beta_{1}+\beta_{1}^{’}\Gamma (EPU_{it};\gamma ,{\bar{EPU}}_{n})\\ +\frac{\partial \Gamma (EPU_{it};\gamma ,{\bar{EPU}}_{n})}{\partial EPU_{it}}(\beta_{1}^{’}EPU_{it}+\beta_{2}^{’}RGDP_{it}+\beta_{3}^{’}FDC_{it}+\beta_{4}^{’}IST_{4it}+\beta_{5}^{’}FEX_{it}) \end{array}$$

Similarly, the influence coefficient $\varphi_{it}$ of control variables on carbon emission can be expressed as:

(10) $$\varphi_{it}=\frac{\partial CO_{2it}}{\partial Z_{it}}=\beta_{i}+\beta_{i}^{’}\Gamma (EPU_{it};\gamma ,{\bar{EPU}}_{n})$$

where $Z_{it}$ stands for one of the control variables related to industrial structure, fiscal expenditure, environmental regulation, economic development level, and financial development condition. According to Equations (3) and (4), there are both linear and nonlinear components of the effects of EPU and control variables on carbon emissions, where β and $\beta^{’}$ stand for linear and nonlinear impact coefficients, respectively.

Considering the complexity of nonlinear models and the dynamics of nonlinear impact relationships, we included the following three steps in analyzing the nonlinear impact of EPU shocks on carbon emissions in China using the PSTR model: first, testing whether the data have significant nonlinear characteristics, and setting the model and parameters; second, applying the nonlinear least-squares (NLS) method; and finally, using the function images to visually compare the threshold effects of EPU on carbon emissions and deeply explore the underlying causes and mechanisms of the nonlinear effects. The empirical analysis detailed in this paper was carried out with the help of MATLAB 2022a software.

### 3.3. Data

#### 3.3.1. Explained Variable

The explained variable in this paper is total carbon emissions at the provincial level in China, and the data are from the “China Emission Accounts and Datasets”, which is compiled by scholars from many research institutions in the United Kingdom, the United States, Central Europe, and other countries, and aims to provide a solid theoretical foundation and technical support for the realization of green and low-carbon development, effectively guaranteeing the accuracy and integrity of the data.

#### 3.3.2. Core Explanatory Variable

Provincial EPU in China serves as the primary explanatory variable in this study. Baker et al. [7] developed an EPU index for China utilizing the English-language newspaper the “South China Morning Post” as the primary study object. This index is currently the most extensively used proxy for EPU globally; however, it has several obvious flaws. First, it relies on textual data from the “South China Morning Post”, a more subjective source when it comes to assessing China’s economic position and policy changes, making it impossible to gauge China’s economic policy uncertainties. Second, because English keywords tend to have simpler interpretations than Chinese keywords when used for screening and indexing, the index cannot capture all the words that signify uncertainty in economic policy. Finally, the index is unsuitable for panel data analysis since it only captures the amount of uncertainty at the national level and ignores the variation within Chinese provinces. Based on this, Yu et al. [33] created an interprovincial EPU index that is very relevant to the Chinese context, overcoming the limitations of the Chinese EPU index by optimizing the target newspaper source, keywords, and construction process. Therefore, this paper uses the Chinese provincial EPU index developed by Yu et al. [33] for the core explanatory variable.

#### 3.3.3. Control Variables

This paper uses the GDP per capita of each province to measure the level of provincial economic development. Furthermore, this paper uses the provincial financial marketability index constructed in the research series of Fan et al. [34] as a proxy for the regional financial development status, which to some extent also responds to the degree of regional financial frictions, and there is an inverse variation relationship between the two. The index measures the degree of financial marketization using the following two basic indices: first, market competition in the financial sector, expressed as the ratio of assets of non-state financial institutions to the assets of all financial institutions; second, marketization of credit fund allocation, expressed as the proportion of liabilities of non-state enterprises to total liabilities. A larger value of the financial marketization index represents a higher level of financial marketization in the region and a likely lower level of financial friction.

For provincial industrial structure, the Thiel index is not only able to reflect the relative weights among the three industries using the output value ratio, but also retains the theoretical basis and economic meaning of structural deviation; thus, an increasing number of studies have adopted the Thiel index to measure the rationalization of industrial structure, and it is widely recognized by academics [35]. In light of this, this study uses the Thiel index to evaluate the level of industrial structure rationalization as a surrogate variable in order to assess the condition of the provincial industrial structure. Its mathematical equation is:

(11) $$TL=\sum_{i=1}^{n}\left(Yi/Y\right)\ln(YiL/YLi)$$

where Y and L denote total output and employment, and i and n denote industries and their sectors, respectively. According to the relevant hypothesis of classical economics, when the economy reaches Pareto optimality, the labor productivity of each industry is equal, or $Y_{i}/L_{i}=Y/L$; then, we have TL=0. Therefore, a greater deviation of the Thiel index from 0 indicates that the industrial structure is more irrational.

This paper uses the general public budget expenditures of each province as a proxy measure for the size of provincial fiscal expenditures. There are many indicators for provincial environmental regulation, the selection of which is based on the research perspective used, as no consensus on the most accurate indicator has yet been reached. Given that the main measure of environmental pollution control in China’s current development stage is administrative regulation, this paper uses the ratio of a completed investment in industrial pollution control to the value added to the secondary industry as a measure of environmental regulation intensity.

The selection of the model indicators used in this study has been introduced. In the specific process of data aggregation, considering the availability and completeness of data, the panel data of 30 Chinese provinces (Tibet, Hong Kong, Macao, and Taiwan are not included due to missing data) from 2004 to 2017 are used as the empirical sample in this paper. Except for the carbon emission data, the original data for each variable were obtained from the “China Economic Network Statistics Database” and “Wind database”. Table 1 displays the descriptive statistics for each variable.

### 3.4. Parameter Estimation

According to the research idea of this paper, we construct the model with EPU indicators as the threshold variables. First, the nonlinear properties of the panel data are investigated, and the transformation function and the number of thresholds is chosen (see Table 2). Furthermore, to reduce the absolute number gap in the data, the total provincial carbon emissions, EPU, financial status index, and fiscal expenditure size are entered into the model in logarithmic form.

The results of the nonlinear tests in Table 2 show that the model statistics all reject the original hypothesis of the linear model at the 5% significance level, confirming that the effect of EPU shocks on carbon emissions in China has significant nonlinear characteristics, while the results of the remaining nonlinear tests all accept the original hypothesis at more than 10% significance; i.e., there is only one optimal transition function of the model (r = 1). Furthermore, based on the RSS, AIC, and BIC values of the model, the optimal number of thresholds is 1 (m = 1). Thus, in this paper, a PSTR model with a transformation function and a threshold was built, and the model’s parameters were estimated using nonlinear least squares, with the results shown in Table 3.

From the estimation results of model parameters shown in Table 3, it can be seen that in the case where provincial EPU is used as the threshold variable, the estimated values of model parameters are all non-zero within the 10% significance level, and the effects of each variable on carbon emissions have nonlinear characteristics. Furthermore, in combining the estimation results of the location parameters and the sample data, it can be seen that the threshold value of EPU is 4.1063, and the number of samples below the threshold value is 91, accounting for 17.95% of the total samples; the number of samples above the threshold value is 416, accounting for 82.05% of the total samples. It can be seen that overall, there is significantly greater distribution of EPU in China’s high-regime provinces than in the low-regime provinces, showing a certain degree of imbalance. In addition, the impact coefficient of provincial EPU shows that its impact coefficient on carbon emissions turns from positive to negative as the level of EPU crosses the threshold value. Moreover, the effects of provincial economic development level, financial development condition, industrial structure, government expenditure scale, and environmental regulation on carbon emissions are all relatively typical nonlinearities.

In summary, the impact of economic policy uncertainty shocks on carbon emissions at the provincial level in China has a significant threshold effect and exhibits complex nonlinear characteristics. The values of the threshold variables show that the degree of economic policy uncertainty in China is still at a higher level in more provinces. The asymptotic transformation of the conversion function smoothly transforms the coefficients of the effects of each explanatory variable on carbon emissions between the two regimes, which lays an important foundation for further in-depth analysis of the nonlinear impact mechanism of economic policy uncertainty shocks on carbon emissions.

## 4. Empirical Results

**Figure 1:**
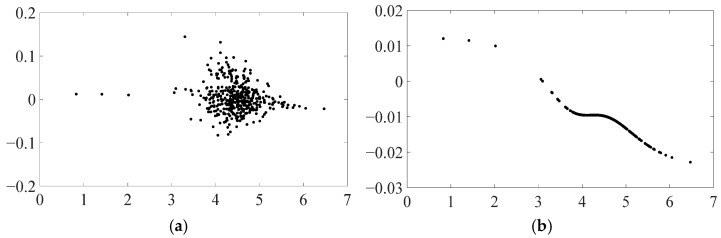
Nonlinear effects of EPU shocks on carbon emissions. Subplots (**a**,**b**) are a scatter plot and function plot, respectively.

**Figure 2:**
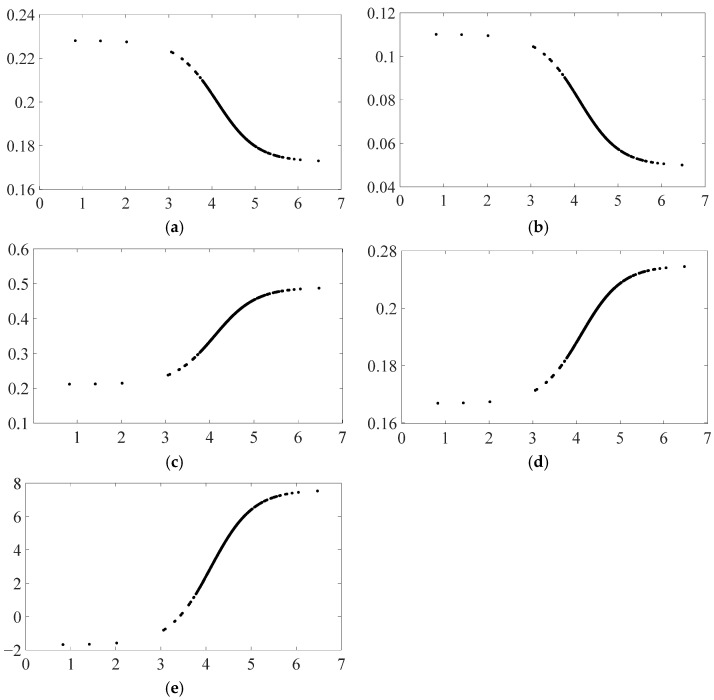
Impact of each control variable on carbon emissions. Subplots (**a**–**e**) show the effects of five control variables on carbon emissions: level of economic development, financial development condition, industrial structure, fiscal expenditure, and environmental regulation, in that order.

To investigate the nonlinear variation mechanism of carbon emissions under the increasing degree of provincial EPU in depth, this paper plots scatter plots and function plots, as shown in Figure 1 and Figure 2, based on the model parameter estimation results and the original data. The scatter plot includes all of the data from 30 provinces, cities, and autonomous regions during the sample period, whereas the function plot fixes each control variable as the mean value of all its sample points, describing the average size of the impact of EPU shocks on carbon emissions. The horizontal coordinate represents the level of EPU, and the vertical coordinate represents the impact of EPU shocks on carbon emissions.

### 4.1. Nonlinear Effects of EPU Shocks on Carbon Emissions

The scatter plot shown in Figure 1a demonstrates that multiple sample points correspond to the same level of EPU and most of them do not overlap, indicating that the direction and magnitude of the effect on carbon emissions may be significantly different in different regions or at different time points in the same region, even if the level of EPU is the same.

In fact, large differences in geographic location, resource endowments, etc., in different regions can be found in the existing literature, such as the level of local economic development, financial development status, industrial structure, government financial support, and environmental regulation, which may lead to different magnitudes and directions of the effects of EPU on carbon emissions. To eliminate the interference of other factors in the model and examine the mechanism of the impact of EPU on carbon emissions more clearly, we fix each control variable in the scatter plot to its respective average level and obtain the function plot shown in Figure 1b. The function plot demonstrates that as the level of EPU increases across the sample, the effect on carbon emissions shifts from positive to negative.

In particular, when provincial EPU is close to the threshold, it has a strong positive impact on carbon emissions; when it is above the threshold, it has a steady negative impact on carbon emissions until stabilizing at a low level. Therefore, it makes intuitive sense that each region will, while taking into account both long-term structural conflicts and short-term economic development goals, have an impact on the attainment of the “double carbon aim” during the economic development process. This is due to the fact that the adjustment of policy programs frequently seeks a dynamic balance among multiple objectives, such as stabilizing growth, modifying structure, and preventing risks, which inexorably results in changes in policy tightness, direction, and strength. As a result, policy uncertainty breeds and influences local carbon emissions.

### 4.2. Impact of Control Variables on Carbon Emissions under Different EPU Regimes

Since both economic and policy consequences of EPU shocks can have an impact on carbon emissions, these two features are chosen as the model’s control variables based on relevant studies. Therefore, for the above identification model of the nonlinear carbon emission effect of EPU, since its setting is influenced by the provincial level of economic development, financial development status, industrial structure, fiscal expenditure scale, and environmental regulation, this section further explores the nonlinear influence mechanism of these five control variables on carbon emission on both sides of the threshold value of EPU, in order to obtain useful conclusions and policy insights regarding the heterogeneity characteristics of the carbon emission effect of EPU. The specific calculation results (calculated based on Equation (10)) are shown in Figure 2.

According to Figure 2, each control variable’s influence on carbon emissions on either side of the EPU threshold value exhibits a clear nonlinear variation pattern. This implies that as provincial EPU rises, control variables also have a nonlinear impact on provincial carbon emissions, significantly altering each variable’s own mechanism of influence. The specific results shown in Figure 2a–e show that the effects of provincial economic and financial development status on carbon emissions are positive in general, and the positive carbon emission effects of economic and financial development status gradually decrease as EPU increases, indicating the improvement of provincial economic and financial development status has a facilitating effect on carbon emissions, while an increase in EPU reduces it. The effects of rationalizing provincial industrial structure and increasing the scale of fiscal spending on carbon emissions are also overall positive and show a gradual expansion of changes around the threshold of EPU, which is diametrically opposed to the characteristics of changes in the carbon emission effects of economic development level and financial development status. The effect of environmental regulation on carbon emissions is negative when EPU is low, but this negative reduction effect gradually changes to a positive pull effect as EPU increases.

### 4.3. Simulation Analysis of Nonlinear Carbon Emission Effects of EPU under Different Values of Control Variables

**Figure 3:**
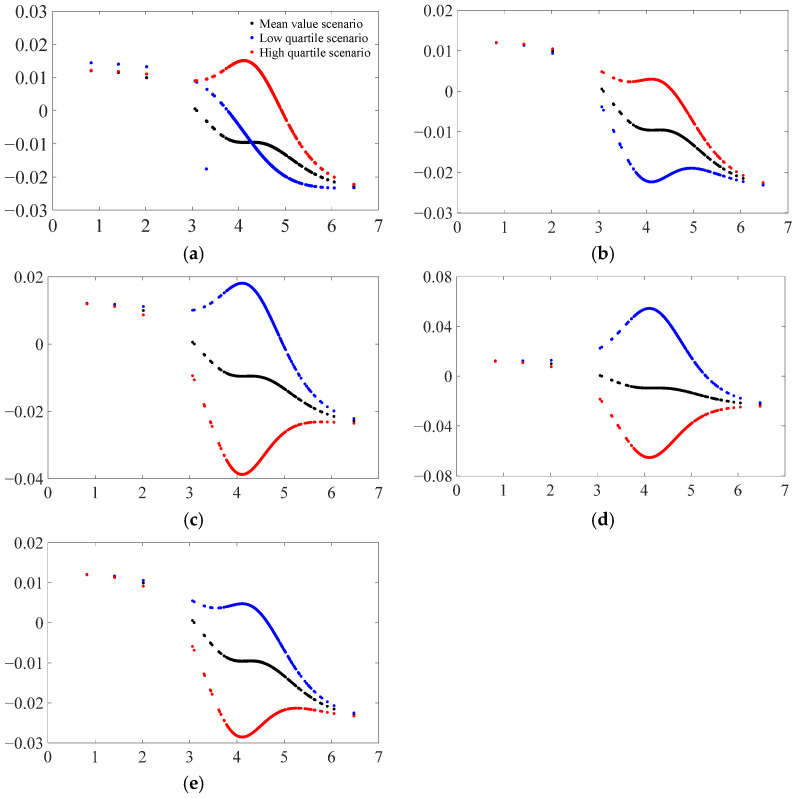
Nonlinear carbon emission effects of EPU under different values of control variables. Subplots (**a**–**e**) show the results for the control variables, level of economic development, financial development status, industrial structure, government spending, and environmental regulation, in that order.

The results for the nonlinear effects of control variables on carbon emissions under different regimes of EPU indicate that there is significant heterogeneity in the cross-sectional characteristics (e.g., level of economic development, financial development status, and environmental regulations) across provinces, and that there are large differences in the carbon emission effects of different cross-sectional characteristics. So how do these cross-sectional factors play a role in the carbon emission effects of EPU? Using Equation (9), this section attempts to control the values of the model control variables and plots the function of the effect of EPU on carbon emission shocks (see Figure 3) to further investigate the mechanism of heterogeneity under nonlinear effects of EPU. The following two scenarios are specifically simulated: first, the target control variable is taken below the mean (10% quantile) and the remaining control variables are taken at the mean; second, the target control variable is taken above the mean (90% quantile) and the remaining control variables are taken at the mean. In addition, as a comparison, in Figure 3, we plot the nonlinear average effect of EPU shocks on carbon emissions for each control variable taking the mean value, which is also shown in Figure 1b.

As can be seen from Figure 3, the nonlinear relationship between EPU and carbon emissions does indeed exhibit a distinct pattern of heterogeneity; i.e., the nonlinear relationship between provincial EPU and carbon emissions subsequently exhibits significant variation in the presence of variations in the values of the control variables. Specifically, from Figure 3a–e, it can be seen that the better the provincial economic and financial development conditions (high quartile), the greater the effect of EPU on carbon emissions; this indicates that the nonlinear carbon emission effect of EPU becomes more significant as provincial economic and financial development conditions improve; in other words, the improvement in provincial economic and financial development status strengthens the carbon emission effect of EPU before and after the threshold. Conversely, the nonlinear carbon emission effects of EPU are greater for poor industrial structure, smaller fiscal expenditures, and poorer environmental regulations (lower quartile).

## 5. Discussion

Since the reform and opening up, China’s energy consumption and carbon dioxide emissions have increased along with the country’s rapid economic growth, and ecological pollution and environmental management are currently faced with a number of major issues. As the domestic and international economic environment and the COVID-19 pandemic continue to change, China’s economic development is facing significant pressure and uncertainty [3,4,5]. In weighing and adjusting various economic objectives, the problem of EPU inevitably becomes prominent. In this context, it is crucial to examine the effect of EPU on carbon emissions and its underlying mechanism, and the solution to this problem may contribute to the realization of China’s “double carbon objective”.

Furthermore, to clarify the current state of research on the topic of this paper and to optimize the research proposal, in the process of combing through studies related to this paper, we found that there is a clear disagreement among academics about the carbon emission effects of EPU, which may even present very different research conclusions. For this reason, we argue that economic and financial factors such as economic structure and economic and financial development status may lead to significant changes in the mechanism of the effect of EPU on carbon emissions, and the effect of EPU shocks on carbon emissions is likely to have nonlinear characteristics. Considering the structural differences of China’s regional economies, we finally used the PSTR model to investigate the effect of EPU on carbon emissions and its mechanism of action.

It was found that the magnitude and direction of the impact of EPU shocks on carbon emissions in China are not constant, but show a positive and then negative pattern of change as EPU increases; moreover, the increase in the level of EPU, in addition to leading to a significant change in the mechanism underlying its own impact on carbon emissions, also triggers a nonlinear migration of the effect of other variables on carbon emissions, which to some extent also explains the existence of a large amount of heterogeneity among Chinese provinces. In provinces with higher levels of economic and financial development, the positive carbon emission effects of EPU are more pronounced; conversely, provinces with lower levels of economic and financial development are more negatively affected by EPU. In contrast, the nonlinear carbon emission effects of EPU are greater in provinces with irrational industrial structures, smaller fiscal expenditures, and poorer environmental regulations.

We argue that the reason behind the above empirical phenomenon is strongly related to the transmission channels through which EPU shocks affect carbon emissions, as described in past relevant studies, and thus the nonlinear effects caused by EPU shocks on carbon emissions at the provincial level in China are mainly due to the differences in their economic and policy effects. Specifically, in terms of economic effects, EPU not only affects policy effectiveness but can also have a nonlinear effect on economic growth [36,37,38]; based on this, when the EPU is low or declining, the provincial economy grows rapidly due to, for example, increased policy effectiveness [39], which has a pulling effect on carbon emissions; conversely, an increase in EPU ultimately has a dampening effect on carbon emissions by inhibiting economic growth dynamics and thus reducing energy consumption. In terms of policy effects, the emission reduction effect of environmental regulation is more effective when EPU is low, but when EPU is high, local governments focus more on stabilizing economic development and overcoming the adverse effects of economic fluctuations, the intensity of environmental regulation may be relaxed, and the emission reduction effect of environmental regulation is weakened or even ineffective. By combining these two effects, it is easy to understand the nonlinear effect of EPU shocks on carbon emissions; the final response dynamics of carbon emissions may depend on the actual magnitude of the relationship between the two effects at different periods and different levels of policy uncertainty. The analysis in Section 4.2 and Section 4.3 of this paper provides an intuitive understanding of the causes and mechanisms of action.

Obviously, the findings of this paper differ from the empirical findings of Jiang et al. [15], Wang et al. [17], Chen et al. [18], and Liu and Zhang [19], because we examined the dynamic impact of provincial EPU on carbon emissions in China from a nonlinear perspective, taking into account the complexity and structural differences of the Chinese provincial economic system. The findings are more consistent with Chinese economic reality, which is the paper’s main marginal contribution. Of course, this paper also has obvious limitations. The analysis of the nonlinear carbon emission effects of EPU lacks a theoretical model to regulate the interpretation, which will be the focus of further research. This paper can also provide a direct empirical basis for the study of related theoretical mechanisms. In addition, in the context of rapid global economic transformation, the impact of EPU has penetrated various fields of production and life, and this study can provide some empirical support and policy inspiration for the correct understanding of environmental risks brought by EPU and the optimization of macroeconomic multi-objective regulation practices.

## 6. Conclusions

Based on the in-depth consideration of issues in China’s economic and environmental development, this paper adopts the PSTR model to systematically investigate the nonlinear impact effects of provincial EPU shocks on carbon emissions and their mechanism of action. The findings demonstrate that the magnitude and direction of the impact of EPU shock on China’s carbon emissions are not constant and exhibit a nonlinear pattern of firstly positive and then adverse changes with an increase in EPU; in addition, an increase in the level of EPU not only causes a significant change in the mechanism underlying its own impact on carbon emissions, but also causes a nonlinear migration of the effects of local economic and financial development, industrial structure, government expenditure, and environmental regulation on carbon emissions; further studies reveal that these cross-sectional factors are important causes and mechanisms of action for the nonlinear carbon emission effects of EPU at the provincial level in China. In conclusion, our study not only confirms the existence of a nonlinear link between EPU and carbon emissions at the provincial level in China, but also sheds some light on the mechanisms and causes underlying their empirical occurrence. These findings provide, on the one hand, direct empirical evidence that improves the understanding of the carbon emissions problem in the context of high EPU, and on the other, an important empirical basis for theoretical exploration of the transmission mechanism of shocks to carbon emissions from EPU. According to the research conclusions, the policy implications of this paper are as follows:

First, although the rise in EPU can curb carbon emissions, it does not achieve green and sustainable economic development at the expense of economic development vitality, which is not in line with the requirements of “high-quality development” of the economy. Therefore, the government should emphasize authoritative interpretation when policy programs are adjusted or major emergencies occur, prevent misjudgment and overreaction, enhance risk management and expectation management capabilities in complex situations, improve the stability of the interaction structure among economic individuals, control economic policy uncertainty within a moderate space from the root, and create an effective policy environment for green and sustainable economic development.

Second, although the decline in EPU creates a good environment for economic development, rapid economic growth at the expense of environmental quality is not in line with the requirements of “high-quality development”. Therefore, the government should actively encourage enterprises to research and develop green technology, promote the further development of their energy structure, and promote “environmentally friendly” economic development with the use of clean energy and the advancement of green technology. In addition, the government should not relax its environmental regulations and improve and perfect the market information disclosure system to encourage enterprises to fulfill their social responsibility.

Third, the difference in regional economic structure has a significant adjustment effect on the carbon emission effect of EPU shock. Therefore, the government should also actively improve the market economic system, optimize the structure of the economic system, afford full play to the market’s ability to self-correct and engage in spontaneous regulation, and reduce the market’s over-reliance on economic policies.

## Figures and Tables

**Table 1:** Descriptive statistics of model variables.

Variable	Mean	Std. Dev	Min	Max
CO_2_	275.181	189.938	872.906	16.462
EPU	98.448	56.920	646.643	2.285
RGDP	34,674.382	22,908.315	136,172	4244
FDC	7.014	2.028	12.660	1.890
IST	0.255	0.157	0.895	0.0175
FEX	2906.611	2300.578	15,037.480	123.020
ERE	0.00397	0.00321	0.0245	0.000307

**Table 2:** Nonlinearity test, residual nonlinearity test, and determination of the number of thresholds.

Statistics	Nonlinearity Test H0: r = 0, H1: r = 1		Residual Nonlinearity Test H0: r = 1, H1: r = 2	
	Value	*p*-Value	Value	*p*-Value
LM	15.555	0.016	10.644	0.101
LMF	2.461	0.024	1.612	0.143
LRT	15.850	0.015	10.880	0.109
**Statistics**	**Determination of the number of thresholds**			
	m = 1		m = 2	
RSS	4.580		4.502	
AIC	−4.425		−4.416	
BIC	−4.281		−4.281	

Note: H0 refers to the null hypothesis and H1 refers to the alternative hypothesis; r = 1 indicates that the model has one transformation function, and similarly, r = 2 indicates that the model has two transformation functions; m = 1 indicates that the model has one location parameter, and similarly, m = 2 indicates that the model has two location parameters.

**Table 3:** Results of model parameter estimation.

Coefficients of Explanatory Variables						Smoothing Parameter
$\beta_{1}$	$\beta_{2}$	$\beta_{3}$	$\beta_{4}$	$\beta_{5}$	$\beta_{6}$	γ
0.0123 *	0.2281 ***	0.1102 ***	0.2114 *	0.1737 ***	−1.6750 *	21.516
**Coefficients of the Transfer Function**						**Location Parameter**
$\beta_{1}^{’}$	$\beta_{2}^{’}$	$\beta_{3}^{’}$	$\beta_{4}^{’}$	$\beta_{5}^{’}$	$\beta_{6}^{’}$	${\bar{EPU}}_{n}$
−0.0364 *	−0.0554 **	−0.0605 *	0.2779 **	0.0959 ***	9.2615 *	4.1083

Note: ***, **, and * represent significance at the 1%, 5%, and 10% levels, respectively.

## Data Availability

The raw/processed data required to reproduce these findings cannot be shared at this time, as the data also form part of an ongoing study.
